# Direct acute respiratory distress syndrome after gastric perforation caused by an intragastric balloon: a case report

**DOI:** 10.1186/s12871-020-01101-y

**Published:** 2020-07-25

**Authors:** Nils Theuerkauf, Tobias Weismüller, Carsten Weißbrich, Jens-Christian Schewe, Christian Putensen, Christian Bode

**Affiliations:** 1 Department of Anesthesiology and Critical Care Medicine, University Hospital Bonn, Venusberg-Campus 1, 53127 Bonn, Germany; 2 Department of Internal Medicine I, University Hospital Bonn, Bonn, Germany

**Keywords:** Chest imaging, Pneumonia, ARDS, Obesity treatment, Abscess, Extracorporeal membrane oxygenation

## Abstract

**Background:**

Acute respiratory distress syndrome (ARDS) is a life-threatening condition and the identification of the underlying direct (pulmonary) or indirect (non-pulmonary) cause is mandatory for a successful treatment. Intragastric balloon (IGB) therapy is a minimal invasive and supposedly harmless option to reduce body weight for the growing number of obese people. We present a case of a young patient who developed a direct ARDS due to initially undiagnosed abdominal pathologies caused by an IGB therapy.

**Case presentation:**

A 23-year old woman was admitted because of a direct ARDS for extracorporeal membrane oxygenation (ECMO) therapy. Weeks before, an IGB has been removed because of abdominal pain and free intraabdominal air. Diagnostic work-up of free intraabdominal air, previous pain of the left shoulder and newly developed abscess pneumonia revealed a perforation of the posterior wall of the gastral antrum. This resulted in a left subphrenic abscess with destruction of the diaphragm, development of pneumonia per continuitatem and subsequent direct lung injury. The gastric perforation was endoscopically clipped and the ARDS was successfully treated under ECMO therapy.

**Conclusion:**

This case illustrates that a patient presenting with direct ARDS may have upper abdominal pathologies caused by a rare complication of a supposedly harmless treatment.

## Background

Acute respiratory distress syndrome (ARDS) is a heterogeneous entity in the setting of an underlying disease that is normally caused by either direct injury to the lung (e.g. aspiration of gastric contents, pneumonia) or indirect injury to the lung (e.g. abdominal sepsis, pancreatitis) [1, 2]. To apply a successful therapeutic regimen in patients with ARDS, the identification of the underlying cause is crucial [2].

Obesity is a major risk factor for numerous chronic diseases including cardiovascular diseases and cancer [3]. Because of its minimally invasive nature, intragastric balloon (IGB) treatment is an upcoming and supposedly harmless option for the more than 1.9 billion obese adults worldwide [4]: Serious adverse events are rare and include migration in 1.4% of patients and gastric perforation in 0.1% [5].

Here we report the development of direct ARDS that is initially caused by gastric perforation after previous IGB therapy.

## Case presentation

**Fig. 1:**
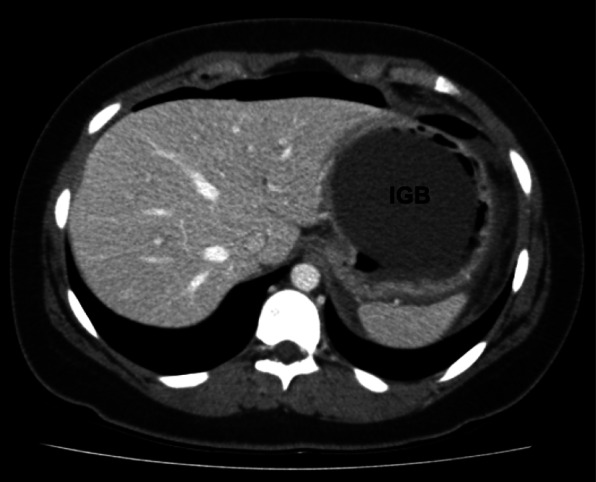
CT-scan of the upper abdomen with free abdominal air and indwelling intragastric balloon. IGB, intragastric balloon

Six weeks before transferal to our university hospital for treatment of direct ARDS, the patient was admitted to a local hospital due to epigastric pain during indwelling of a 6-month-old IGB. The IGB therapy led to a total weight loss of 5 kg body weight with a reduction of the body mass index from 29.7 to 27.9. After diagnosing free abdominal air (Fig. 1), the IGB was removed by an outpatient endoscopy. No further diagnostics or treatment were performed.

**Fig. 2:**
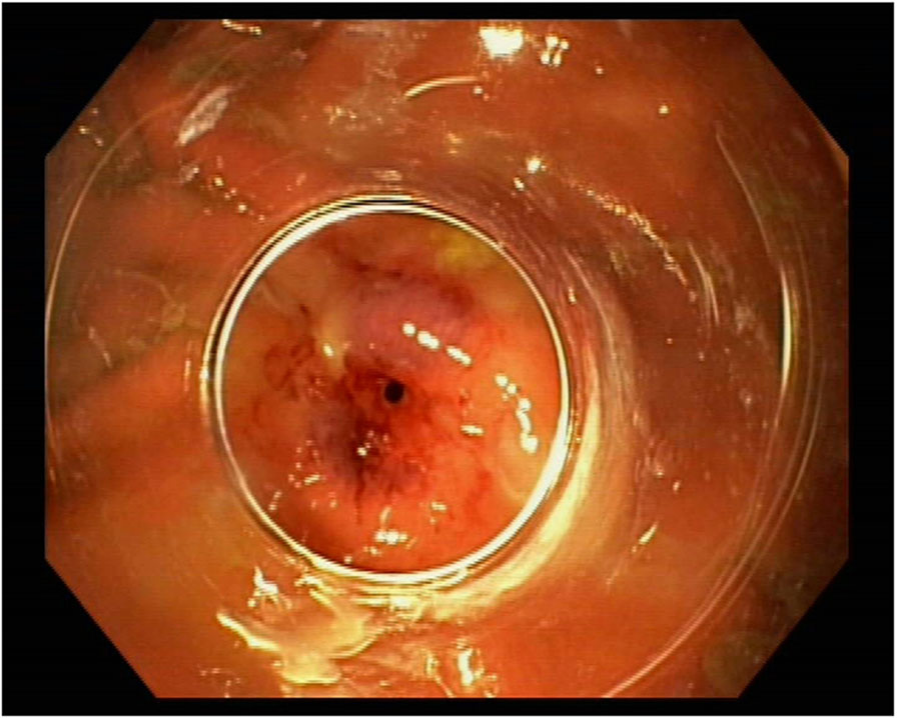
Perforation of the posterior wall of the gastral antrum was endoscopically diagnosed

**Fig. 3:**
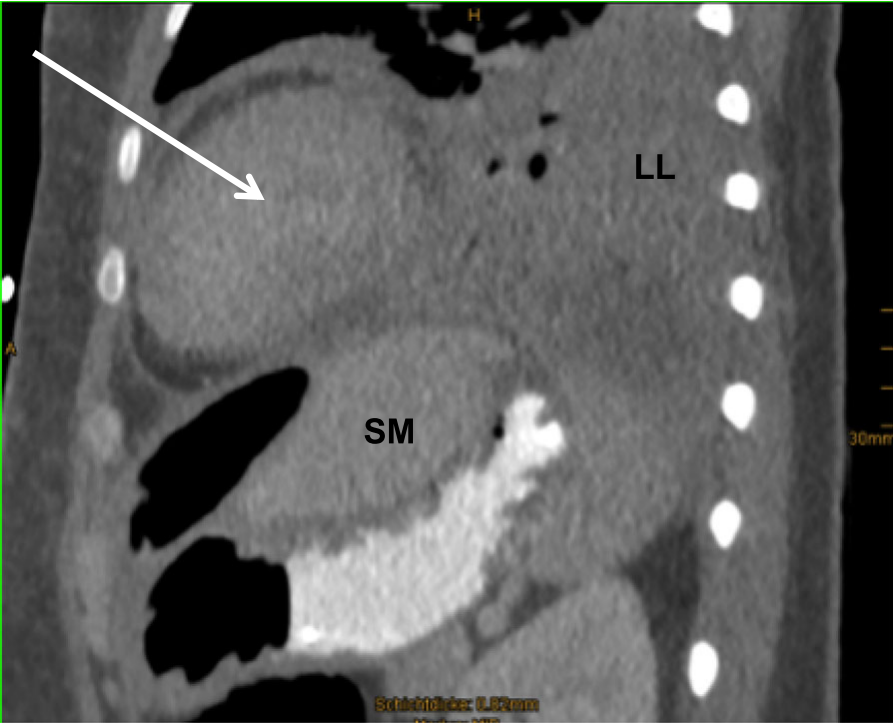
CT-scan demonstrating left subphrenic abscess with destruction of the diaphragm and development of pneumonia per continuitatem. LL, left lower lobe; SM, stomach; white asterisk: subphrenic abscess

Following removal of the IGB, the patient developed increasing pain of the left shoulder. Under the suspicion of subacromial bursitis, the patient was treated with corticosteroids for a period of 10 days. Several days later, the patient became symptomatic with progressive dyspnea. CT-scan of the chest revealed pneumonia with abscess of the left lower lobe. Due to rapidly deteriorating hypoxemic lung failure the patient necessitated orotracheal intubation and mechanical ventilation. Based on a PaO_2_/FIO_2_ ratio of 86 mmHg at PEEP-level of 10 mbar and peak inspiratory pressure of 28 mbar within 12 h after intubation, the patient was presented to our hospital for evaluation of veno-venous extracorporeal lung support (vvECMO). Diagnostic work-up of previous free intraabdominal air, pain of the left shoulder and pneumonia with abscess in a young, otherwise immunocompetent patient led to the diagnosis of a perforation of the posterior wall of the gastral antrum (Fig. 2), resulting in a left subphrenic abscess with destruction of the diaphragm and development of pneumonia per continuitatem (Fig. 3). With proof of both, gastric perforation and staphylococcus subspecies in the abscess drainage, empiric antibiotic treatment with piperacilline / tazobactame, clarithromycine and cefazoline was changed to caspofungin, vancomycin and cefazolin. The gastric perforation could be visualized endoscopically and successfully be closed by use of an over-the-scope-clip. During a repeated CT-scan, a pigtail drainage was percutaneously inserted under radiological guidance and was used as a suction-irrigation drainage. This drainage allowed timely resolution of the infradiaphragmatic abscess. Despite rapid diagnosis of the underlying disease process and despite successful endoscopic closure of the perforated stomach, advanced destruction of the left-sided diaphragm and alveolar spaces of the left lower lobe led to persistent, extensive air-leakage and finally inadequate alveolar ventilation. Beside lung-protective ventilatory strategies with high PEEP, inverse ratio ventilation and low tidal volumes, the subsequent progressive hypoxemic lung failure necessitated mechanical support by means of bifemoral vvECMO for a period of 15 days and subsequently further mechanical ventilation to maintain adequate oxygenation. Due to the underlying extensive air-leakage, adjunctive therapy strategies such as inhaled nitric oxide or prone positioning have not been attempted. ARDS was successfully treated and the patient was discharged in a good clinical condition and without any neurological sequel after six weeks.

## Discussion and conclusions

Direct and indirect ARDS can be considered as different diseases that are characterized by different pathophysiological, radiological and mechanical patterns [6]. ARDS from direct causes including pneumonia and aspiration is initiated by an insult of alveolar epithelium while ARDS from indirect causes such as abdominal sepsis is triggered by endothelial damage [1]. Yet, current report demonstrates the development of pneumonia with subsequent direct ARDS due to an undiagnosed abdominal infection. Therefore, even when pneumonia was considered as the main trigger of ARDS, the abdominal abscess might also have contributed to diseases progression by endothelial injury. Mixed etiologies of lung injury have been described before and are often related to trauma-associated ARDS through chest injury and systemic inflammatory response syndrome [2, 7].

To our knowledge this is the first ARDS caused by a supposedly harmless IGB treatment. Gastric perforation by IGB occurs in only 0.1% of treatments [4]. Furthermore, the clinical diagnosis of GI tract perforation is challenging as the symptoms may be non-specific [5]. This combination might be the reason for the delayed diagnosis of gastric perforation and development of an abdominal abscess in the current case. Our patient presented with i) pneumonia of the left lower lobe and ARDS ii) gastric perforation and iii) left subphrenic abscess. Given that the patient underwent IGB treatment, followed by epigastric symptoms and pain in the left shoulder, the perforation of the abscess into the lung is the most likely cause for the pneumonia per continuitatem and subsequent direct ARDS. Consistent with this, it has been shown that in 44% of subphrenic abscesses the chest findings dominated the clinical picture while in 42% of the cases the abdominal findings were most prominent [8].

In conclusion, this report demonstrates that patients with direct ARDS may have additional upper abdominal pathologic conditions as risk factors including abscesses [2]. Although pneumonia exists and could have explained the patient’s lung failure alone, careful anamnesis and clinical diagnostic led to the correct diagnosis of direct ARDS after gastric perforation caused by an IGB. In addition to a complete anamnesis, we recommend comprehensive diagnostics including both thoracic and abdominal CT-scan in every admission to minimize the possibility of an additional non-pulmonary septic focus in patients with assumed direct ARDS (and vice versa).

## Data Availability

Data sharing not applicable to this article as no datasets were generated or analysed during the current study.

## Abbreviations

ARDS: Acute respiratory distress syndrome
ECMO: Extracorporeal membrane oxygenation
IGB: Intragastric balloon
